# Non-Instrumented DVA: Assessment of Performance and Clinical Feasibility in Children Ages 2 Through 13 Years

**DOI:** 10.3390/children13040456

**Published:** 2026-03-26

**Authors:** Cathey P. Norton, Nancy S. Darr, Mary Katherine Beshears, Katherine Catalano, Tyra Dillard, Mahayla J. K. Gamble, Magdalene Olerich, Sadie Rodell Rupp

**Affiliations:** School of Physical Therapy, Belmont University, 1900 Belmont Boulevard, Nashville, TN 37212, USA; cathey.norton@belmont.edu (C.P.N.); mary.beshears@bruins.belmont.edu (M.K.B.); katherine.catalano@bruins.belmont.edu (K.C.); tyra.dillard@bruins.belmont.edu (T.D.); mahayla.gamble@bruins.belmont.edu (M.J.K.G.); magdalene.bollinger@bruins.belmont.edu (M.O.); sadie.rodell@bruins.belmont.edu (S.R.R.)

**Keywords:** dynamic visual acuity, DVA, vestibulo-occular reflex, VOR, vestibular dysfunction, children, development, balance, academic performance, pediatric

## Abstract

**Highlights:**

**What are the main findings?**

• Non-instrumented DVA testing is feasible for children aged 3 years and older and can be used to identify vestibular dysfunction across age groups.
• Performance on non-instrumented DVA testing does not differ significantly across age groups, with most children demonstrating results comparable to established adult normative values.

**What are the implications of the main findings?**

• The non-instrumented DVA can be administered to children using a simple methodology by newly trained clinicians and represents a promising and clinically feasible screening tool for identifying children with possible vestibular dysfunction. While most 3-year-olds can perform DVA testing, some may present challenges due to difficulty reading LEA Symbols or other behavioral factors.
• This tool shows promise as a feasible clinical option that may be integrated into pediatric physical therapy practice to assist in referral recommendations.

**Abstract:**

**Background/Objectives:** Vestibular disorders can have functional consequences for children, including balance and gross motor delays, academic difficulties and behavioral manifestations; however, they are frequently undiagnosed in children. The purposes of this study were to evaluate the feasibility and clinical utility of performing a non-instrumented dynamic visual acuity (DVA) test as a primary screening tool for children and to examine typical performance on this non-instrumented DVA test in a large sample of children ages 2 through 13 years. **Methods:** A clinical DVA test was administered to a convenience sample of 208 children aged 2–13 years. Static visual acuity was assessed using a standard Snellen or LEA eye chart, depending on the child’s ability to read letters. Dynamic visual acuity was then measured while the examiner manually rotated the child’s head at 2 Hz (240 bpm). DVA was calculated as the number of lines of visual acuity lost with head movement. **Results:** All children aged 4 years and older and 67% of 3-year-olds successfully completed DVA testing. Most 2-year-olds and 33% of 3-year-olds were unable to complete DVA testing. Although the number of visual acuity lines lost with rotational head oscillations at 2 Hz varied between age groups, Kruskal–Wallis test indicated no significant difference in DVA scores between age groups (K = 12.721, DF = 9, P = 0.176). Ninety percent of children who were able to perform DVA testing lost two or fewer lines of visual acuity with head rotations consistent with adult norms. **Conclusions:** This method of DVA testing is an easily accessible and promising clinically feasible screening tool for identifying children with vestibular dysfunction. The authors recommend widespread vestibular screening of children to facilitate rapid referral for diagnosis and treatment of children with vestibular dysfunction.

## 1. Introduction

The vestibulo-ocular reflex (VOR) is essential for stabilizing gaze during head movements by enabling the eyes and head to move in equal and opposite directions. When the VOR is impaired, the eyes cannot adequately stabilize gaze during head motion, resulting in blurred vision and unstable fixation while moving. The dynamic visual acuity (DVA) test evaluates VOR function by measuring an individual’s ability to see clearly while the head is in motion [1]. Poor performance on the DVA test may indicate VOR impairment consistent with underlying vestibular dysfunction, along with central problems such as cerebellar dysfunction [2].

Vestibular dysfunction in children is frequently under-recognized, thereby potentially contributing to delays in diagnosis. In part, this under-recognition stems from children’s difficulty with articulating complex symptoms accompanied by vestibular disorders [3]. Unlike adults, children experiencing visual disturbances commonly associated with vestibular dysfunction may not raise complaints, as they are often unaware that a problem exists [4]. Additionally, symptoms may present as difficulty focusing during school or subtle balance and gross motor challenges, leading parents to assume the child is simply uncooperative or uncoordinated [3]. When subtle presentations occur alongside achievement of gross motor developmental milestones such as functional ambulation, clinicians may overlook the possibility of an underlying vestibular etiology [5]. Ultimately, delayed recognition and consequent late diagnosis of vestibular dysfunction in children can be attributed to the culmination of communication limitations, nonspecific symptom presentation, and developmental milestone attainment that masks underlying vestibular involvement. Despite estimates on the prevalence of pediatric vestibular disorders ranging from 0.7% to 15%, it has been shown that less than 20% of affected children are correctly diagnosed within one month of the onset of symptoms, and a large majority of affected children experience diagnostic delay exceeding one year [6].

Symptoms resulting from vestibular dysfunction can precipitate significant functional limitations, including increased risk of falls, impaired reading ability, and reduced participation in physical activities, with delayed diagnosis further complicating these issues [7,8,9]. Children with sensorineural hearing loss and concurrent vestibular impairment often show delays in motor skills and difficulty maintaining gaze stability, particularly when visual or somatosensory input is challenged, as well as deficits in sensory organization [4,9]. Without effective vestibular input during critical periods of childhood, typical postural control may not develop, leading to progressive motor delays. Vestibular dysfunction may also serve as a hidden barrier to learning. Franco and Panhoca [7] found a significant association between vestibular abnormalities and poor school performance and acknowledged that vestibular dysfunction can disrupt the sequential eye fixations required for reading, directly interfering with the development of fundamental academic skills. Moreover, common childhood conditions such as otitis media with effusion can have lasting vestibular consequences. Pazdro-Zastawny et al. [10] demonstrated that children with a history of middle ear effusion exhibited increased postural sway and nystagmus up to five years after treatment. Their findings underscore the potential for undetected dysfunction to persist and affect motor development. Beyond physical and academic consequences, pediatric vestibular disorders may impact psychosocial development. Children with vestibular disorders exhibit higher rates of emotional and behavioral difficulties, including anxiety, depression, and attention problems [11,12], and are at increased risk for cognitive and psychiatric comorbidities such as learning disabilities and developmental delays, highlighting the vestibular system’s integral role in neurodevelopment [13]. Under-identified vestibular dysfunction leads to prolonged, untreated symptoms, compounding these challenges and diminishing social participation and quality of life [6]. Collectively, these findings highlight the urgent need for accessible, cost-effective screening tools to facilitate early identification and intervention.

Several advanced diagnostic tools exist for evaluating vestibular dysfunction in children, including Videonystagmography (VNG), Rotary Chair Testing, Video Head Impulse Test (vHIT), and Vestibular Evoked Myogenic Potentials (VEMPs). While these instrumented tests provide comprehensive diagnostic data, they are costly and require specialized equipment and advanced clinician training. Consequently, such resources are often unavailable and inaccessible in many pediatric clinical settings [6,14,15]. Thus, these tests may be impractical as first-line screening tools, and vestibular dysfunction frequently goes undetected or is diagnosed late, contributing to prolonged symptoms and developmental consequences. Barriers such as limited time for screening have also been reported by pediatric rehabilitation professionals, further reducing the utilization of vestibular assessments as screening tools within clinical practice [15,16]. This gap underscores the need for accessible, efficient screening methods to identify children who warrant referrals for comprehensive vestibular evaluation. In response to these barriers, standardized clinical balance tests such as the Pediatric Balance Scale (PBS), pediatric Modified Clinical Test of Sensory Interaction in Balance (mCTSIB) and Timed Up and Go (TUG) have been recommended as low-cost alternatives for assessing functional balance in children [15]. However, these tools primarily evaluate functional balance and postural control rather than directly assessing vestibular system function, thereby potentially limiting comprehensive evaluation critical for pediatric patients with balance deficits [17]. Ultimately, vestibular function screening is underutilized within pediatric settings, resulting in missed opportunities for early detection and intervention [17]. The non-instrumented DVA presents an accessible and practical screening tool for vestibular system function that enables clinicians to quickly and accurately rule out vestibular dysfunction. Broader adoption of this screening tool could improve the timely identification of vestibular pathology and optimize developmental outcomes.

Among available vestibular tests, the DVA test is a clinically relevant option for assessing vestibular function in adults, demonstrating strong diagnostic validity and reliability for the instrumented version and promising results for the non-instrumented version. Herdman et al. [1] established the instrumented (computerized) version of DVA testing as a highly reliable test for assessing VOR function in adults as it ensured head velocities were kept within a range that isolated VOR from other eye-tracking systems. Testing ultimately demonstrated high sensitivity and specificity when differentiating between adults with vestibular deficits versus those in the control group [1].

Given the excellent reliability and diagnostic validity of the instrumented version of DVA testing, subsequent research on non-instrumented DVA testing has been conducted to determine the utility of DVA testing that accommodates more accessible and less expensive methods [18,19,20,21]. Early research from Longridge and Mallinson [18] established strong predictive parallels between the “Dynamic Illegible E” test (DIE), a version of non-instrumented DVA testing, and the instrumented caloric testing, a gold standard diagnostic test for unilateral vestibular hypofunction. The authors ultimately reported a positive correlation (*p* < 0.05) between the two tests as the degree of abnormality within DIE testing increased with the degree of caloric reduction. Because all control group participants lost no more than one line on the DIE test, the authors defined a DIE cutoff score as a loss of more than two lines. Furthermore, Roberts and Gans [19] reported non-instrumented horizontal-plane DVA testing as more sensitive towards VOR impairments than non-instrumented vertical-plane testing, with an overall moderately high negative predictive value of 83.6%. Recent studies report the “bedside” applicability of non-instrumented DVA testing for detecting VOR impairment in adults [21] and its high clinical feasibility, as the test is fast, inexpensive, and accessible [20]. Overall, while variability in testing protocol has limited the consistency of reported reliability and validity measures, the non-instrumented DVA test has been widely recognized as a clinically feasible screening tool for identifying adults with possible vestibular dysfunction.

Although research remains limited, studies indicate that DVA testing serves as a clinically feasible method for assessing vestibular function in children. Early work by Rine introduced a simple, non-instrumented DVA test using LEA symbols, demonstrating excellent test–retest reliability (ICC = 0.94) and 100% sensitivity and specificity for detecting bilateral vestibular hypofunction [4]. This study confirmed DVA testing as appropriate for children as young as three years old. Subsequent research reinforced the clinical effectiveness of DVA testing, reporting high sensitivity for identifying vestibular hypofunction in children and establishing minimal detectable change scores (8 optotypes), which enable clinicians to monitor meaningful progress over time [22]. The non-instrumented DVA test for children is both cost-effective and time-efficient, requiring only a vision chart, a chair, and a metronome, compared to more involved and costly diagnostic tests. It also demonstrated strong diagnostic accuracy with a negative likelihood ratio of 0.18 for ruling out vestibular hypofunction [22], thereby allowing clinicians to quickly and accurately rule out vestibular hypofunction. Together, these findings provide strong evidence for the construct validity and reliability of non-instrumented DVA testing as an accessible, cost-effective screening tool that can guide referral decisions and complement diagnostic evaluations in pediatric settings.

Adult norms for normal DVA performance, clinically defined as fewer than two lines of visual acuity loss [18], have been reported in the literature. Using the computerized (instrumented) NIH Toolbox DVA (cDVA) test, Li et al. conducted a large (n = 3992) cross-sectional study across ages 3–85 years and established normative reference values [23]. Results showed a median cDVA difference of 0.070–0.080 logMAR (≈1 line of visual acuity loss during head movement) for adults ages 18–49. In pediatric populations, Rine and Braswell demonstrated through use of a non-instrumented clinical approach that children as young as three years typically show two or fewer lines of visual acuity loss during head movement, which is considered within normal limits [4]. The discrepancy in reporting metrics, logMAR difference versus lines of visual acuity lost, highlights a lack of standardization in normative data, indicating a need for further research. Despite this variability, normative adult DVA benchmarks are clearly defined in the literature, helping to serve as a reference for DVA performance interpretation in other populations.

Although comprehensive diagnostic tests are valuable, their cost, instrumentation, and specialized training requirements make the tests hard to access and impractical as first-line screening in routine pediatric care. In contrast, non-instrumented DVA testing directly analyzes VOR function with favorable reliability, sensitivity, and practical feasibility in children [4,22]. Despite its promising applicability in pediatric settings, research on the usage of the non-instrumented DVA as a pediatric screening tool is limited, underscoring a need for further investigation of its utility, cost-effectiveness, and overall practicality in pediatric populations. The purposes of this study were to evaluate the feasibility and clinical utility of performing a non-instrumented DVA test as a primary screening tool for children and to examine typical performance on this non-instrumented DVA test in a large sample of children ages 2 through 13 years.

## 2. Materials and Methods

### 2.1. Participants

A convenience sample of 208 children (91 males and 117 females) aged 2 through 13 years was recruited from schools, churches, preschools, and other community organizations. All children were developing typically per parent report. Study inclusion criteria included the ability to follow simple verbal instructions and consistently identify either shapes on the LEA symbols chart (Good-Lite Company, Elgin, IL, USA) or letters on the Snellen eye chart (PECULA, Shenzhen, China). Children were excluded from study participation if parents reported any neurologic or developmental health conditions, seizures within the previous 6 months, or any other physician-recommended activity limitations that would prohibit safe participation in the study.

### 2.2. Rater Training

Twenty-nine raters (27 entry-level physical therapy students and 2 physical therapy faculty) participated in data collection. All raters received formal training in the administration and scoring of the DVA by two Belmont University Physical Therapy Faculty members, both of whom are board-certified clinical specialists in Neurologic Physical Therapy. Each student rater completed a training course that included demonstration and guided practice in administering and scoring the DVA with peers and non-participant children. All data collection occurred under the direct supervision of two faculty mentors.

### 2.3. Materials and Equipment

This study utilized a non-instrumented version of the Dynamic Visual Acuity (DVA) test [4,24]. Visual acuity was assessed using a Snellen eye chart [25] for children who were able to identify letters and a LEA symbol chart [26] for children unable to read letters. Equipment required for DVA testing included a chair with back support and appropriate height to ensure the child’s feet were supported flat on the floor, along with a tape measure, an eye chart, and a metronome.

### 2.4. Procedures

Prior to participation, each parent completed written informed consent and a health history form on behalf of their child. Participants aged 7 to 13 years also provided written assent using an age-appropriate child assent form. Parents signed a waiver of assent for children aged 6 and under. A preschool-level script describing the study was read to these young children before they were asked for verbal assent. All procedures and consent forms were approved by the Belmont University Institutional Review Board (IRB #1281). After obtaining informed consent and age-appropriate assent, each participant was assigned an identification number to ensure confidentiality throughout testing and data management.

The testing protocol was standardized across all sites to minimize variability. All testing was conducted individually, with two examiners assisting with testing each child. Faculty mentors were also present during testing. The investigators confirmed the date of birth, absence of exclusion criteria, and the child’s ability to follow simple instructions with the parent before initiating testing. Two children were excluded from the study. One child had remarkable atypical oculomotor function and was subsequently referred to her pediatrician with a recommendation for neuro-ophthalmology evaluation. Visual acuity could not be assessed in the other child because she did not bring her glasses and could not read the Snellen chart without them.

Test administrators then confirmed each child’s ability to identify letters or shapes to determine the appropriate eye chart. If children could not quickly and easily read letters, then the LEA symbols chart was used, and if they could not consistently identify letters or LEA symbol shapes, they were disqualified from participation. A reference card displaying the LEA symbols was used prior to testing to ensure the participant could identify each symbol. Children were not disqualified if they were consistent in how they mislabeled LEA symbols shapes. For example, many children consistently mislabeled the “apple” as a heart or the “house” as a triangle. Participants who normally wore glasses or contact lenses wore them during testing.

Each child was positioned in an appropriately sized chair with a back that enabled the child to sit with their back supported and their hips and knees at 90 degrees of flexion with their feet flat on the floor. The chair was placed at the appropriate distance from the eye chart per protocol (10 feet from the LEA symbols chart, 20 feet from the Snellen chart) with the chart affixed to the wall at the child’s eye level. Testing was conducted individually with two testers assisting with DVA testing. One rater (R1) stood behind the child, and a second rater (R2) was positioned by the eye chart.

Binocular static visual acuity was assessed first and recorded in logMAR units, with the lowest line read with two or fewer errors documented. Two trials of dynamic visual acuity with head rotations were then performed. R1 positioned the child’s head in 30 degrees of cervical spine flexion and passively rotated their head horizontally right and left 20–30 degrees (approximately 15 degrees to each side) in a sinusoidal pattern at a frequency of 2 Hz, guided by a metronome set to 240 beats per minute. Head rotation at 2 Hz was selected based on previously published pediatric and adult DVA protocols demonstrating that this frequency provides adequate VOR challenge while remaining safe. Consistency was supported through structured rater training, faculty oversight, and metronome pacing, though some amplitude variability inherent in manual methods is acknowledged [8]. R2 was positioned next to the eye chart and used a piece of white paper to cover the lines immediately below the line being read by the child to help maintain visual focus. R2 also verified accuracy of the child’s responses as they read each line on the eye chart. The lowest line the participant could read, with two or fewer errors documented during head movement, was recorded. The number of lines lost between static and dynamic conditions was calculated and documented as the DVA score. Each child completed one practice session followed by two test trials. A small number of children refused a second trial of DVA with head movement.

## 3. Results

**Figure 1:**
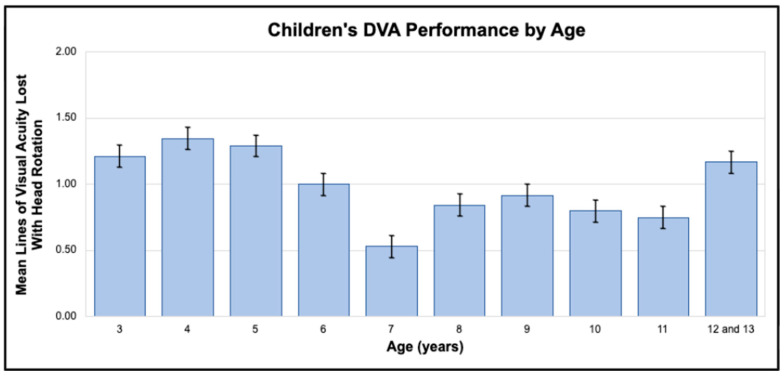
Means and standard deviations for lines of visual acuity lost with head rotation at 2 Hz.

**Figure 2:**
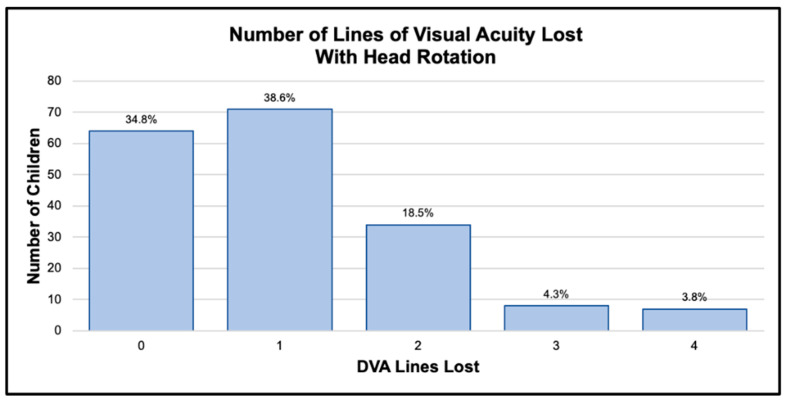
Numbers and percentages of children obtaining each score on DVA testing.

Children were divided into 12 groups in one-year age increments for descriptive data analyses. The 12- and 13-year-old groups were subsequently combined for remaining statistical analyses due to relatively small numbers in each group (7 and 5, respectively). Number of lines of visual acuity lost with head rotations was used for all data analyses. Of the 208 children, 185 were able to successfully complete DVA testing (Table 1). Only one of the 15 two-year-olds was able to complete DVA testing; results for this child were not used for further data analysis. Two-thirds of 3-year-olds and all children four years and older were able to successfully complete DVA testing. Although mean lines lost with head rotations differed slightly between age groups (Figure 1), a Kruskal–Wallis test indicated no significant difference in DVA scores between age groups (K = 12.721, DF = 9, P = 0.176). Ninety percent of children who were able to perform DVA testing lost two or fewer lines of visual acuity with head rotations consistent with adult norms [23] (Figure 2).

## 4. Discussion

The purpose of this study was to evaluate the clinical utility of the non-instrumented Dynamic Visual Acuity (DVA) test as a primary screening tool for vestibular dysfunction in pediatric populations and to examine the typical DVA performance in a large sample of children aged 2 to 13 years. The results of this study indicate that the non-instrumented DVA can be administered to children using a simple methodology by newly trained clinicians and yields outcomes comparable to those reported in both instrumented [23] and non-instrumented DVA studies [4].

All children aged 4 and older completed DVA testing. Two-year-old children were largely unable to complete the test, indicating limited feasibility in this age group. All but one two-year-old and one-third of three-year-olds were unable to complete DVA testing. Although two-thirds of 3-year-olds were able to complete DVA testing, many required more time and patience from the examiner to complete the test. The most common limitation to DVA testing in these children was the inability to consistently identify the LEA symbols. Children were not required to accurately name the LEA symbols but had to be consistent with the names they chose for the symbols. For example, some young children referred to the “apple” symbol as a “heart” and the “square” symbol as a “triangle.” Some two- and three-year-olds also refused to let the examiner shake their head during the dynamic portion of DVA testing. Comparisons across studies are difficult due to variations in protocol and scoring.

No significant differences in DVA performance were found across age groups, suggesting consistency across childhood. Most children (92%) in this study, who successfully completed DVA testing, lost two or fewer lines of visual acuity, which is consistent with previously reported adult norms on instrumented [23] and non-instrumented clinical DVA [18]. Health histories of the 15 children who successfully completed DVA testing but lost 3 or more lines of visual acuity (DVA ≥ 3) with head rotations were further examined for commonalities and differences between this group and the remaining 170 children who lost two or fewer lines of visual acuity (DVA ≤ 2). Twelve parents reported health conditions that could be potentially associated with atypical vestibular function or balance (Table 2). The most common of these reported conditions was ear infections. Adverse effects of ear infections on vestibular function and balance in children have been well documented in the literature [27,28,29]. Thirty-three percent of children in the DVA ≥ 3 group had ear infections in the year prior to testing, and 67% had a lifetime history of ear infections. Rates of ear infections for the DVA ≤ 2 group were 38% in the previous year, with 53.5% of children in this group having a lifetime ear infection history. While it appears that children in the DVA ≥ 3 group may have a higher lifetime rate of ear infections, all information about ear infection history was provided via parent report. Parents were asked to report how many ear infections their child had in the past year and whether the child had experienced any ear infections before that period. They were not asked about ear infection effusion or severity which limits comparison of DVA performance in children with and without ear infection history. This examination of health history in the DVA ≥ 3 group should be considered exploratory based on the following factors: (1) small sample size, (2) health history was obtained by parent report and (3) vestibular dysfunction was not confirmed with instrumented testing. Future studies should include prospective examination of DVA performance in children who have current documented ear infections with follow-up DVA testing to examine potential long-term effects of ear infections on vestibular function. Finally, the authors recommend consideration of targeted DVA testing for children with ear infection history both in clinical settings and in future studies where vestibular dysfunction may influence study outcomes.

Two children aged 5 years and older (22%) in the DVA ≥ 3 group had parent-reported Attention Deficit/Hyperactivity Disorder (ADHD), which was very similar to the parent-reported rate (20%) in the DVA ≤ 2 group (Table 2). The authors chose to only include children ages 5 years and older in these calculations, as ADHD is often not diagnosed until school age. Although the relationships between ADHD have not been examined extensively, Gur-Hartman et al. found that children with diagnosed ADHD demonstrated evidence of vestibular hypofunction on DVA testing using a method very similar to that of the current study [24]. Children with ADHD also had reduced balance ability compared to peers without ADHD as measured by 9 items on the Bruininks–Oseretsky Test of Motor Proficiency (BOT-2). These differences were even more pronounced in children with ADHD and a comorbid diagnosis of developmental coordination disorder (DCD). Although the current study demonstrated similar rates of ADHD in children with typical and atypical DVA results, it should be noted that the sample sizes were small. Additionally, all information about ADHD diagnoses was provided via parent report and not corroborated with diagnostic testing.

The non-instrumented DVA test serves as a highly feasible and practical screening tool for pediatric populations, primarily due to its minimal equipment requirements and low cost. Rine & Braswell [4] and Christy et al. [22] described DVA testing methods for clinical settings. Both found clinical DVA testing to be reliable and able to accurately identify children with vestibular dysfunction. Unlike advanced diagnostic measures such as Videonystagmography or Rotary Chair Testing, which are often inaccessible in pediatric settings, the non-instrumented DVA requires only a LEA and/or Snellen vision chart, a chair, and a metronome. The simplicity of the equipment required allows the test to be successfully administered across a wide variety of environments; in this study, the DVA was administered in therapy gyms, daycare centers, gymnastics facilities, churches, and private homes. DVA testing requires less than 10 min per child to administer, overcoming a major barrier in pediatric care, where clinicians may bypass vestibular screening due to schedule constraints [30]. By providing accurate results in diverse settings without the need for expensive, advanced technology, the non-instrumented DVA offers an efficient solution for identifying children with vestibular hypofunction [4]. The current study successfully utilized a similar method to that first described by Rine & Braswell [4] and more recently by Gur-Hartman [24] to assess DVA in children aged 3 through 13 years. It is unique in that entry-level physical therapy students administered the DVA to a large sample of children, following a structured training protocol. This indicates that novice clinicians can become proficient at DVA administration with minimal training.

The non-instrumented DVA uniquely contributes to pediatric vestibular screening because it directly assesses an individual’s ability to use VOR to maintain visual stability during head movement without specialized equipment or training; the DVA test additionally provides diagnostic information not captured by functional balance testing. By offering a rapid way to identify visual–vestibular deficits, the DVA can guide timely referral and facilitate earlier initiation of targeted vestibular rehabilitation, ultimately supporting improved functional outcomes.

A primary limitation of non-instrumented DVA testing relates to variability across methodologies, which complicates direct comparison of results. Although standardized head movements at approximately 2 Hz are a shared requirement, studies differ in optotype choice, scoring approach, and termination criteria. Also of note is the possible head movement variability of non-instrumented DVA testing. Despite these inconsistencies, research consistently demonstrates that reduced DVA performance is associated with underlying VOR impairment [4,18,24]. The simplified protocol in this study (i.e., minimal equipment, short testing duration, high feasibility for novice and experienced clinicians alike) enhances clinical practicality but may reduce sensitivity relative to instrumented assessments, particularly for detecting subtle changes over time. Future investigators may wish to add head-mounted sensors to monitor head amplitude variability. While this may improve accuracy, it will add to the cost, which may be prohibitive and decrease feasibility in many clinics. Nevertheless, its efficiency and diagnostic relevance reinforce its value as an efficient first-line screen to identify children who may benefit from further vestibular rehabilitation.

## 5. Conclusions

Non-instrumented DVA testing is a clinically feasible and accessible screening tool for children aged 3 years and older, with performance levels that demonstrate consistency across age groups and align with established adult norms [23]. Its minimal equipment requirements and the ability for novice clinicians to achieve proficiency make it an efficient first-line assessment suitable for diverse pediatric clinical settings. Implementing screening can facilitate earlier identification of vestibular dysfunction, particularly in children with contributing health histories like ear infections, thereby guiding timely referrals for comprehensive evaluation [17]. Widespread adoption of the non-instrumented DVA test has the potential to optimize functional and developmental outcomes through the initiation of targeted vestibular rehabilitation [5,24].

## Figures and Tables

**Table 1:** Identifies numbers and percentages of children successful with DVA testing and descriptive statistics for this group.

Age (years)	Total Number	Children Successful with DVA Testing		Lines of Visual Acuity Lost with Head Rotation			
		n	%	Mean	SD	95% CI Lower Bound	95% CI Upper Bound
2	15	1	6.7				
3	28	19	67.9	1.21	0.23	0.75	1.67
4	29	29	100	1.35	0.19	0.97	1.72
5	31	31	100	1.29	0.18	0.93	1.65
6	19	19	100	1.00	0.24	0.53	1.47
7	16	16	100	0.53	0.25	0.04	1.02
8	19	19	100	0.84	0.23	0.38	1.30
9	12	12	100	0.92	0.29	0.34	1.50
10	15	15	100	0.80	0.26	0.28	1.32
11	12	12	100	0.75	0.29	0.17	1.33
12&13	12	12	100	1.17	0.29	0.59	1.75

**Table 2:** Provides detailed information for children who lost 3 or more lines of visual acuity.

Child Number	Age	Lines Lost	Ear Infections in Last Year	Evidence of Ear Infections in Last Year or Prior	Parent Comments
**1**	3	4	yes	yes	Skull fracture, feeding therapy, reflux, 2 sets of ear tubes
**2**	3	3	no	no	
**3**	4	4	no	no	Resolved gross motor delays
**4**	4	4	yes	yes	Recurring ear infections, ear tubes
**5**	4	3	yes	yes	2 sets of ear tubes
**6**	4	3	no	no	
**7**	5	4	no	no	Behavioral health, speech therapy
**8**	5	4	no	no	
**9**	5	3	yes	yes	ADHD
**10**	5	3	no	yes	
**11**	5	3	no	yes	Ear tubes at 1.5 years old
**12**	6	3	no	yes	
**13**	8	4	yes	yes	
**14**	8	3	no	yes	
**15**	10	4	no	yes	ADHD, motion sickness
**Total**			5	10	

## Data Availability

Data are unavailable to others due to privacy restrictions enforced by the United States government (HIPAA, FERPA). The protocol for this study approved by Belmont University Institutional Review Board does not include permission for data sharing. The authors are willing to collaborate with others who would find this research valuable.
